# Individual differences in speed–accuracy trade-off influence social decision-making in dyads

**DOI:** 10.1098/rspb.2025.1077

**Published:** 2025-07-16

**Authors:** Kiri Kuroda, Alan N. Tump, Ralf H. J. M. Kurvers

**Affiliations:** ^1^ Center for Adaptive Rationality, Max Planck Institute for Human Development, Berlin 14195, Germany; ^2^ Japan Society for the Promotion of Science, Chiyoda-ku, Tokyo 102-0083, Japan; ^3^ School of Sociology, Kwansei Gakuin University, Nishinomiya, Hyogo 662-8501, Japan; ^4^ Exzellenzcluster Science of Intelligence, Technische Universität Berlin, Berlin 10623, Germany; ^5^ Robert Koch Institute, Berlin 13353, Germany; ^6^ Leibniz-Institute of Freshwater Ecology and Inland Fisheries, Berlin 12587, Germany

**Keywords:** speed–accuracy trade-off, social information, drift–diffusion model, social interaction, conformity, group decision-making

## Abstract

Speed–accuracy trade-offs are a fundamental aspect of decision-making, requiring individuals to balance collecting more information against making faster decisions. Although speed–accuracy trade-offs have been studied at the individual level, their role in human decision-making in social settings remains poorly understood—even though faster, and possibly more error-prone, decisions often have more social influence than slower decisions. We examined how individual differences in speed–accuracy trade-off preferences shape decision-making in pairs, using an interactive online experiment and drift–diffusion modelling. Participants first performed a perceptual task alone, allowing us to estimate their individual drift rates and decision thresholds, the key cognitive determinants of speed–accuracy trade-off preferences. They then performed the task in pairs, sharing decisions in real-time. Pair accuracy depended on the faster (and thus more error-prone) member, and not on the slower (but more accurate) member. Social decisions were not worse than individual ones because faster members increased their thresholds in the social condition and became more accurate, while slower members incorporated less social information. These findings show that individuals adjusted their social information use to the speed–accuracy trade-off preferences of their partners, highlighting the importance of such individual differences for understanding social behaviour.

## 1. Introduction

A fundamental challenge for individuals during decision-making is the trade-off between speed and accuracy. When making decisions, individuals need to continuously choose between collecting more information, which generally leads to better decisions, and forgoing more information in order to make faster, but possibly more error-prone, decisions [1]. An individual foraging under predation risk [2,3] who invests little time in detecting predators may reap higher foraging rewards, but at the expense of a higher predation risk. Alternatively, they may invest more time in detecting predators, leading to more accurate predation detection, but at the expense of lower foraging returns. Evidence for such speed–accuracy trade-offs has been found across a wide range of animal species, including bees [4], ants [5], fish [6] and monkeys [7], as well as in humans (see [8] for a review), across decision contexts such as perceptual [9], multisensory [10] and sample-based decisions [11].

Previous research on the role of speed–accuracy trade-offs in human decision-making has been largely limited to the individual level (but see [3] for a review on nonhuman gregarious animals and speed–accuracy trade-offs). Yet there is ample evidence that individuals consistently differ in how they navigate speed–accuracy trade-offs (e.g., [9,12–14]). This means that in group decisions, individuals may be confronted with others with different speed–accuracy trade-off preferences and need to resolve possible resulting conflicts. Furthermore, a range of studies have shown that decisions made earlier by members of a group (i.e. social information) have a strong influence on the other group members [15–19]. Such social information can even cause information cascades, where later-deciding individuals ignore their private information and follow the decisions of early-deciding individuals [20]. When the speed–accuracy trade-off preferences of individuals in groups differ, individuals who prefer speed over accuracy may therefore be the first individuals to make decisions (see also [21]). This, in turn, may lead to poorer decisions in groups. This prediction is somewhat at odds with most research on human group decision-making, which typically predicts benefits of social information exchange (e.g. [22,23]). However, few studies incorporate the natural timing of decisions; instead, they use round-based paradigms in which all individuals decide independently before exchanging information [18].

When individuals experience others’ decisions in real-time, additional information is available that may change the collective dynamics. For example, group members may weight social information as a function of how quickly it arrives [24] and/or its accuracy [25–27]. Moreover, individuals could adjust the timing of their decisions in light of other group members’ tendencies. These and other mechanisms could, therefore, mitigate a drop in accuracy in groups in which members with different speed–accuracy trade-off preferences share decisions in real time.

**Figure 1.**
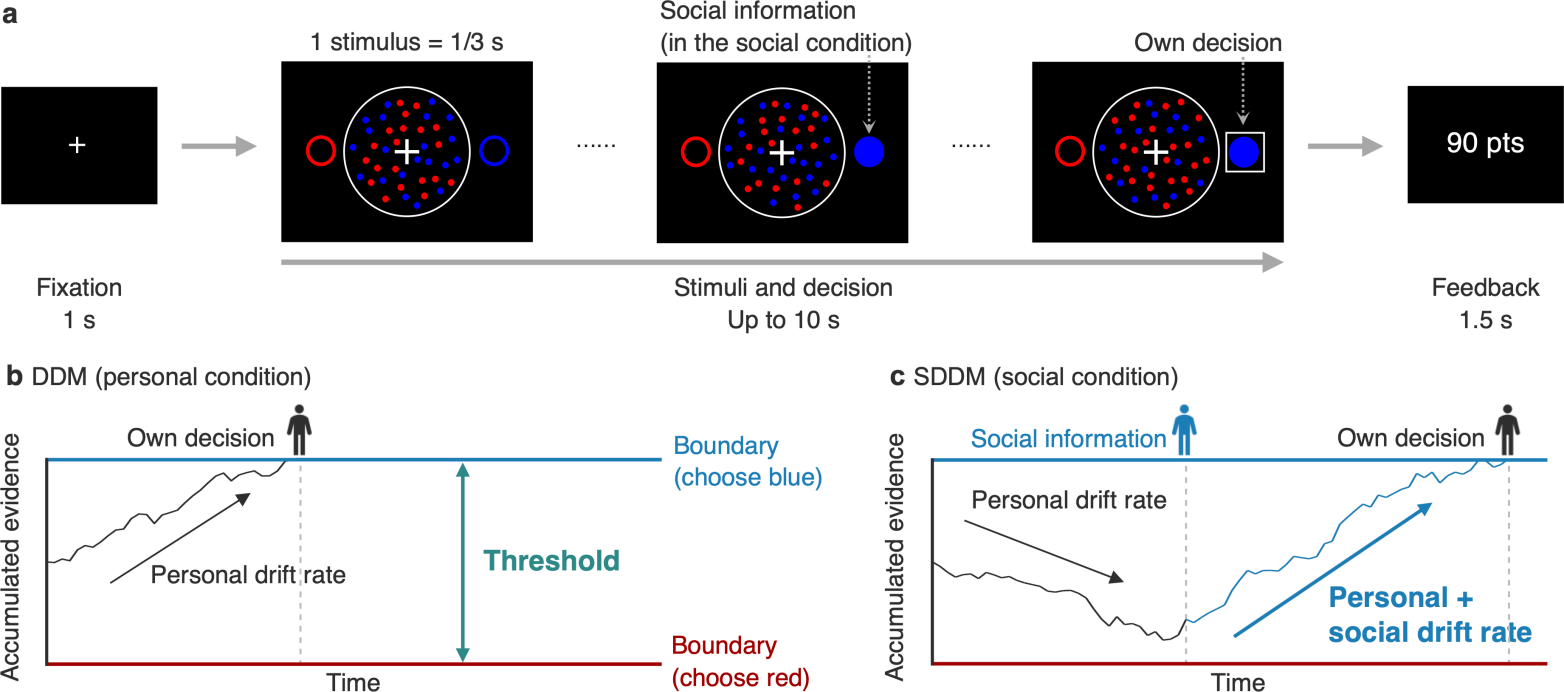
Task and drift–diffusion models (DDMs). (a) Trial flow. (b) Illustration of the DDM. (c) Illustration of the social DDM (SDDM) with two individuals. Non-decision time is omitted from panels (b) and (c) for simplicity.

To understand the cognitive dispositions that make pair members leaders or followers in decisions featuring speed–accuracy trade-offs, the way in which pair members use social information from leaders, and how social information affects pair accuracy, we conducted a binary perceptual decision-making task with real-time information exchange (figure 1a). First, participants conducted the task alone, allowing us to quantify individual differences in speed–accuracy trade-off preference. They then conducted the same task while paired randomly with another participant, with real-time exchange of decisions. Participants’ behaviour was analyzed using the drift–diffusion model (DDM) [28], which allowed us to estimate participants’ decision threshold—that is, how much information they require to make a decision—which directly governs their speed–accuracy trade-off preference (figure 1b). We also measured how strongly participants weighted the social information they received (i.e. their partner’s response) by applying a social DDM [15] (SDDM; figure 1c).

We found that pair members with lower decision thresholds were more likely to act as leaders, and that these individuals set an upper bound on a pair’s mean accuracy. That is, the presence of fast (and inaccurate) individuals is detrimental to overall pair accuracy. Pair members with higher decision thresholds (who were therefore more accurate) did not shape overall pair accuracy. We also observed that the pair members with the higher decision threshold weighted social information less than their faster partners did. Using simulations, we show that these adjustments in social information weighting are adaptive when facing an individual with a different speed–accuracy trade-off preference.

## 2. Results

### (a) Task

We recruited 114 participants from Prolific (an online platform for recruiting research participants; https://www.prolific.com/) to perform an online visual perception task (figure 1a) in a personal and a social condition. In the personal condition, participants observed a sequence of dot stimuli containing a total of 40 red and blue dots for 0.33 s and decided, within 10 s, which colour was dominant. Participants received 100 points (= £0.02) for correct responses, with a 2-point penalty for each second taken; they received nothing for incorrect responses. They then were told the correct answer.

In the social condition, participants were randomly paired with another participant; they then performed the task together. The task was identical to the personal condition, except that whenever one pair member made a decision, the other member saw it in real time (‘Social information’ in figure 1a). Pair members viewed the same stimuli and were rewarded only for the accuracy of their own decisions. Removing dropouts and invalid trials resulted in a final sample of 84 participants (see ‘Data exclusion’ in electronic supplementary material for data exclusion criteria, and Methods for full experimental details).

### (b) (S)DDM and regression analyses

We modeled participants’ decision processes in the personal condition using the DDM [28], which assumes that individuals accumulate stochastic evidence toward a decision boundary at a constant rate (i.e. the personal drift rate; figure 1b). The personal drift rate can be interpreted as perceptual sensitivity [29]. When the evidence reaches one of two boundaries (i.e. red or blue), the individual chooses the corresponding option. Hereafter, we refer to the distance between the two boundaries as the threshold. The threshold indicates how much evidence an individual needs to make a decision and it thus governs their speed–accuracy trade-off preference. Higher decision thresholds (i.e. weighting accuracy over speed) are typically interpreted as more conservative decision-making [29].

We modelled participants’ decision processes in the social condition using the SDDM (figure 1c) [15], which has an additional parameter: the social drift rate. This is an additional drift toward the option chosen by the individual’s partner and it thus represents the degree of reliance on social information. See electronic supplementary material, Supplementary Methods (‘Parameter estimation,’ ‘Parameter recovery,’ and ‘Posterior predictive check’), figures S1, S2, and table S1 for details of the analyses and results.

The experimental data were analyzed using Bayesian hierarchical generalized linear (mixed) models. Below we report the median and 95% highest-density interval of posterior statistics of interest (ρ = Spearman correlation coefficient; *r* = Pearson correlation coefficient; *b* = standardized regression coefficient; *M* = mean). See electronic supplementary material, Supplementary Methods (‘Regression analyses’) and table S2 for details of the analyses.

### (c) Individual differences in speed–accuracy trade-off preferences: personal condition

**Figure 2.**
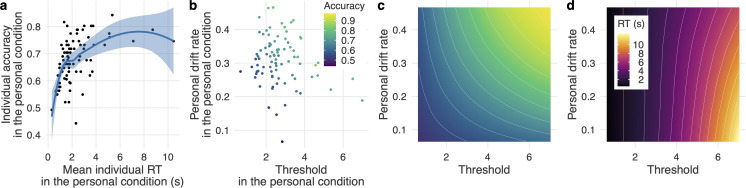
Individual differences in the personal condition. (a) Mean individual response time (RT) and accuracy in the personal condition. Each dot represents one participant. The blue line indicates the locally estimated scatterplot smoothing (LOESS); the shaded area indicates the 95% confidence interval. (b) Participants’ personal drift rates and thresholds in the personal condition. Each dot represents one participant. (c) Predicted individual accuracy. Colours represent predicted individual accuracy from 1000 simulations for each combination of threshold and personal drift rate. See panel b for legend. (d) Predicted mean individual response times. Colours represent the predicted mean individual response time from 1000 simulations.

We first investigated participants’ accuracy and response time in the personal condition. Figure 2a shows a credible positive correlation between an individual’s mean response time and their accuracy in the personal condition (ρ = 0.57 [0.39, 0.76]). This result shows that participants who took more time to decide were also more accurate. There were substantial individual differences in both speed and accuracy of decisions. Participants’ mean response time was 2.27 s; mean accuracy was 67.4%.

As expected, individual accuracy increased as a function of both threshold and personal drift rate (figure 2b; threshold: *b* = 0.06 [0.06, 0.07]; personal drift rate: *b* = 0.07 [0.06, 0.07]). That is, participants with higher thresholds and stronger personal drift rates made more accurate decisions. Individuals with higher thresholds had longer response times (*b* = 1.45 [1.33, 1.56]), whereas the effect of the personal drift rate on response times was not credible (*b* = −0.06 [−0.12, 0.01]; electronic supplementary material, figure S3a). There was no credible correlation between threshold and personal drift rate (figure 2b; *r* = −0.19 [−0.41, 0.03]).

To further investigate the suitability of the DDM for this paradigm, we ran DDM simulations using the ranges of participants’ individual estimates (see ‘DDM simulations to predict accuracy and response time’ in the electronic supplementary material for details). Figure 2c,d show that these DDM predictions were consistent with the empirical results (figure 2b, electronic supplementary material, figure S3a): accuracy increased as a function of both threshold and personal drift rate, whereas response time increased predominantly as a function of threshold. See also electronic supplementary material, figure S3c,d for accuracy and mean response time over trials.

### (d) Pair-level behaviour

#### (i) Equal overall accuracy in personal and social condition

**Figure 3.**
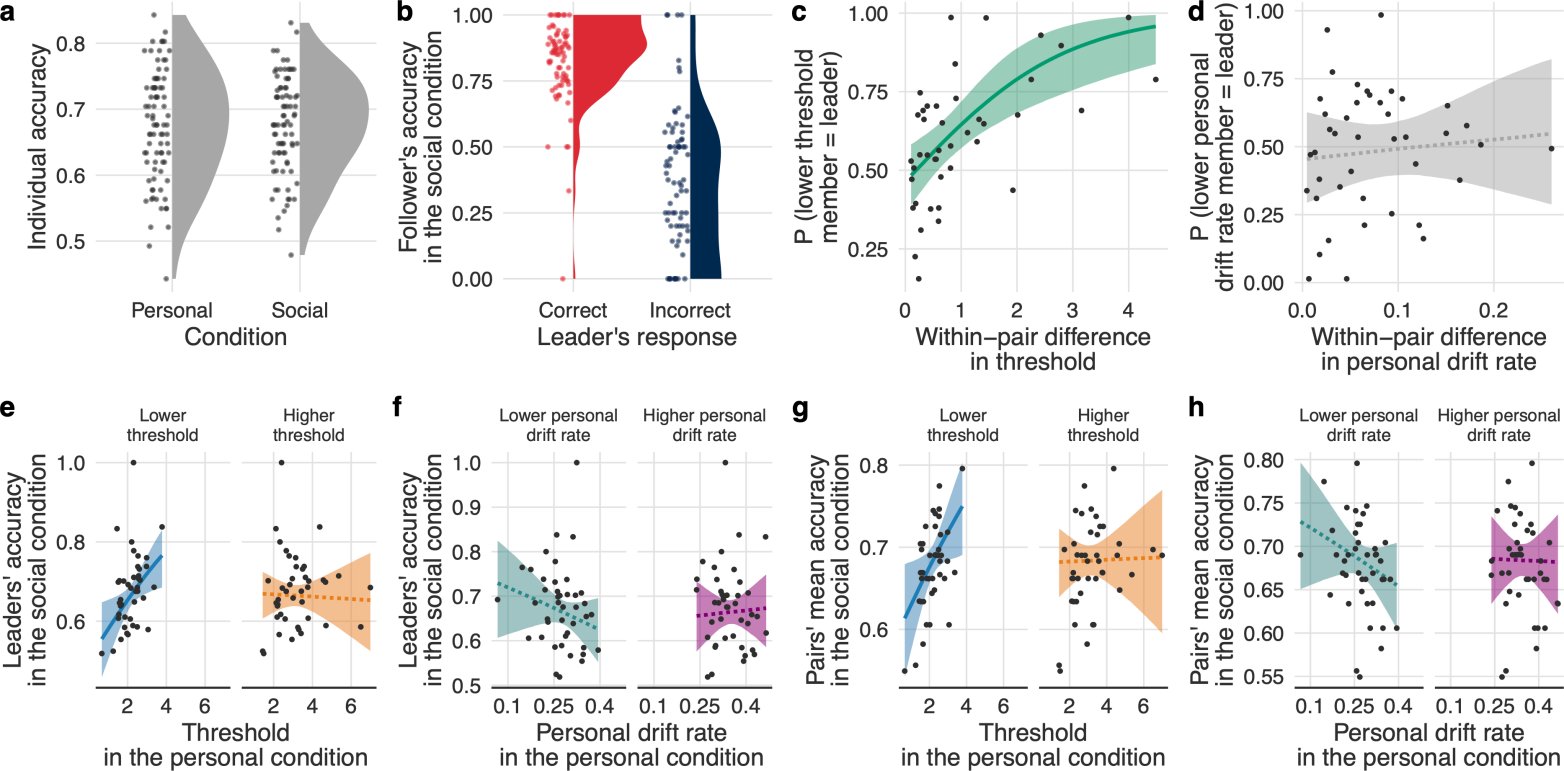
Pairs' mean accuracy and drift–diffusion model (DDM) parameters. (a) Individual accuracy in the personal and social conditions. (b) Accuracy of second decisions in a pair. Each dot indicates a participant but can be based on a different number of underlying trials. (c,d) Probability of being the leader and within-pair difference in (c) threshold and (d) personal drift rate. (e,f) Leaders’ accuracy as a function of higher and lower (e) thresholds and (f) personal drift rates in a pair. (g,h) Pairs’ mean accuracy as a function of higher and lower (g) thresholds and (h) personal drift rates in a pair. In panels c–h, fitted lines indicate the mean conditional effects (solid line = credible effect; dashed line = non-credible effect); shaded areas indicate the 95% uncertainty intervals of the mean.

We next examined whether social coupling improved or worsened accuracy. Figure 3a shows no credible difference in individual accuracy between the personal and social conditions (*b* = 0.01 [−0.10, 0.11]): on average, social coupling was neither beneficial nor detrimental to individual accuracy (see electronic supplementary material, figure S3b for the relationship between response time and accuracy in the social condition).

To check whether social information affected participants’ decisions, we compared the accuracy of the second-deciding member—the follower—when the first-deciding member—the leader—was correct and when they were wrong. Followers’ accuracy was credibly higher when the leader was correct than when the leader was wrong (*b* = 2.11 [1.78, 2.45]; figure 3b). This result suggests that individuals at least partly followed social information.

#### (ii) Less conservative members led pairs

To explore which individual- and pair-level characteristics determined the social decision-making processes—that is, who produced/used social information and who contributed to the pair’s mean accuracy—we first examined the effects of within-pair differences in threshold and personal drift rate on the probability of being the leader in a pair. The within-pair differences in the parameters were based on the personal condition so as to have independent estimates of participants’ personal drift rates and thresholds.

The probability of the pair member with the lower threshold (as estimated from the personal condition) being the leader increased as a function of the within-pair difference in threshold (*b* = 0.76 [0.31, 1.24]; figure 3c). Overall, pairs therefore tended to be led by the member who had collected less perceptual evidence (figure 1b) and thus may have been less accurate (figure 2b,c). Within-pair differences in personal drift rate had no credible effect on the probability of being the leader (*b* = 0.08 [−0.27, 0.50]; figure 3d). These results match the finding from the personal condition that response times (and by extension being the leader in a pair) are mostly governed by thresholds and not by personal drift rates (figure 2d).

Based on these results, we examined what affected the accuracy of the leaders. Given that leaders tended to have the lower threshold in their pair, we expected that the accuracy of the first response in a pair would predominantly be a function of the threshold of the pair member with the lower, not the higher, threshold. As figure 3e shows, the threshold of the member with the lower threshold in a pair had a positive effect on the leader’s individual accuracy (*b* = 0.19 [0.03, 0.33]). In other words, the lower the threshold of the lower-threshold pair members, the worse the first decisions were. The threshold of the higher-threshold member had no credible effect on the accuracy of first decisions (*b* = −0.02 [−0.18, 0.16]). Personal drift rates had no effect on the accuracy of first decisions for either member (lower personal drift rate: *b* = −0.09 [−0.25, 0.07]; higher personal drift rate: *b* = 0.02 [−0.14, 0.18]; figure 3f).

We observed a similar pattern for the mean accuracy of the pairs: pairs’ mean accuracy increased as a function of the threshold of the lower-threshold member (*b* = 0.12 [0.01, 0.23]; figure 3g). There was no effect of the threshold of the higher-threshold member (*b* = 0.01 [−0.12, 0.13]; figure 3g). Similar to leaders’ accuracy, the personal drift rates did not affect pairs’ mean accuracy (lower personal drift rate: *b* = −0.07 [−0.18, 0.04]; higher personal drift rate: *b* = −0.00 [−0.12, 0.11]; figure 3h).

In sum, pairs were led by the members whose decision-making was less conservative (i.e., who had lower decision thresholds), and this affected pairs’ overall accuracy. However, decisions in the social condition were, overall, not worse than decisions in the personal condition (figure 3a). While lower- and higher-threshold members partially maintained their threshold differences in the social condition, we also found evidence in this condition for coordination of thresholds between pair members. That is, the lower-threshold members increased their thresholds in the social condition, whereas the higher-threshold members decreased theirs (see electronic supplementary material, Supplementary Results and figures S4, S5).

#### (iii) Members with lower thresholds had higher social drift rates

We next investigated the social drift rate. The higher an individual’s social drift rate, the more they incorporated social information available from the leader into their evidence accumulation process (see figure 1c and Methods for details of the SDDM).

**Figure 4.**
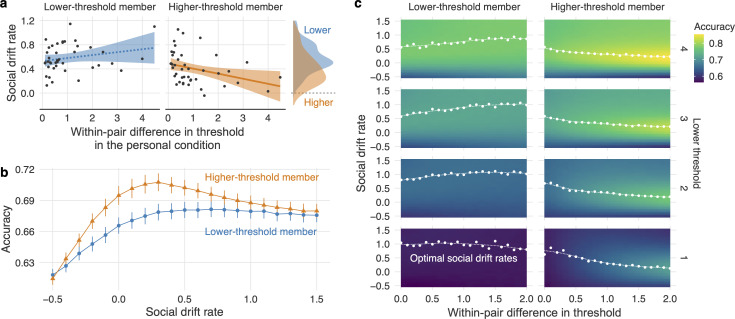
Social drift rate and simulation results. (a) Social drift rates and within-pair differences in thresholds. Fitted lines indicate the mean conditional effects (solid line indicates credible effect; dashed line indicates non-credible effect); shaded areas indicate the 95% uncertainty intervals of the mean. (b) Simulated individual accuracy as a function of social drift rate. Error bars indicate the standard errors of the means. (c) Simulated individual accuracy as a function of within-pair difference in threshold, social drift rate and the lower threshold in a pair. White dots and dashed spline curves indicate the optimal social drift rates (*y*-axis).

Figure 4a shows the social drift rates of participants, separately for pair members with lower and higher thresholds. Both lower- and higher-threshold pair members had credibly positive social drift rates (lower threshold: *M* = 0.58 [0.50, 0.66]; higher threshold: *M* = 0.40 [0.32, 0.48]). Almost all participants followed social information, confirming the result shown in figure 3b (see electronic supplementary material, figure S2g for social drift rates relative to personal drift rates and electronic supplementary material, figure S6b for results of the post-session questionnaire data). Lower-threshold members had credibly higher social drift rates than higher-threshold members (*b* = 0.18 [0.07, 0.29]; see density plot in figure 4a). Thus, although lower-threshold members observed social information less frequently (as they generally made decisions faster), they incorporated social information more when they did observe it. Several explanations may underlie this effect. First, lower-threshold members may learn that their partner takes more time (or is more accurate), hence trust this information more. *Vice versa*, higher-threshold members may start distrusting the faster (and less accurate) responses from their partners. Second, lower-threshold members may have been partly aware that they collected relatively little information prior to making a decision and were, as a result, less confident in their personal information. Third, lower-threshold members may have only responded slowly when they lacked confidence, and they were easily influenced by social information.

In addition, we observed that an increasing within-pair difference in threshold had different effects on the social drift rate of the lower- and higher-threshold members. For higher-threshold members, social drift rates decreased credibly as a function of within-pair difference in thresholds (*b* = −0.09 [−0.16, −0.02]; figure 4a), whereas for lower-threshold members, social drift rates increased, though this was not credible (*b* = 0.05 [−0.02, 0.12]; figure 4a). The interaction effect was credible (*b* = −0.14 [−0.25, −0.04]). In sum, members with higher thresholds in the personal condition (i.e. those who required more information to make decisions, and who thereby generally made better decisions) gave less weight to social information than did their less conservative counterparts. However, since almost all participants had positive social drift rates, the more conservative members were still influenced by the less conservative members (see also figure 3b,g).

#### (iv) Simulations examining the impact of social drift rate on accuracy

To examine how social drift rate impacts individual accuracy for lower- and higher-threshold members, we ran simulations using participants’ SDDM estimates from the social condition. Each agent in a pair was assigned their paired participant’s estimates of threshold, personal drift rate and non-decision time from the social condition, and the 42 pairs from the experiment were simulated. To study the effect of the social drift rate, we varied each agent’s social drift rate from −0.5 to 1.5 in increments of 0.1, covering the empirical range found in the social condition (figure 4a). We performed 10 000 simulations (trials) for each pair and calculated the mean accuracy for each pair member.

Figure 4b shows the simulated accuracy as a function of social drift rates for the higher- and lower-threshold pair members. The accuracy of the lower-threshold members increased with an increasing social drift rate, reaching a ceiling effect at a social drift rate of 0.5. Unlike higher-threshold members, lower-threshold members did not suffer from high social drift rates because their partner’s choices were generally better than their own. Notably, the higher-threshold member is also expected to benefit from using some social information, since their accuracy did not peak at a social drift rate value of 0 but rather at approximately 0.3. This result is in line with theoretical studies showing that even a small weighting of an opinion slightly above chance improves collective accuracy [30]. However, the difference between the maximum possible accuracy (at the optimal level of social drift) and an individual’s accuracy without any social drift (i.e. social drift is 0) was relatively small ( ± 1.5%), implying that substantial accuracy improvements in the social condition are unlikely (see also figure 3a). Finally, in the simulations, the accuracy of higher-threshold members suffered when they relied too heavily on social information. Hence, we expected higher-threshold members to have lower social drift rates than lower-threshold members, as was indeed found in the empirical data (figure 4a).

#### (v) Simulations incorporating the within-pair difference in thresholds

The within-pair differences in thresholds may have modulated the impact of social drift rate on accuracy. In fact, participants tended to adjust their social drift rates as a function of within-pair differences in thresholds (figure 4a). We therefore ran a second simulation to examine the impact of within-pair differences in thresholds on accuracy. We varied the threshold of the lower-threshold member (1, 2, 3, or 4), the within-pair difference in threshold (from 0 to 2 in increments of 0.1) and the social drift rate (from −0.5 to 1.5 in increments of 0.1). All these ranges roughly match the empirical distributions. As in the first simulation, we generated 42 pairs and 10 000 trials for each combination of the three parameters. We used individuals’ estimates from the social condition for agents’ personal drift rate and non-decision time.

We can draw three observations from the results of the simulations shown in figure 4c. First, the colour of subpanels gets brighter from bottom to top, regardless of whether agents had higher or lower thresholds. This pattern indicates that agents became more accurate as their thresholds increased (see also figure 2c). Second, for pair members with lower thresholds (left column), the optimal social drift rates (white dots and lines) lie between 0.5 and 1.0 regardless of their own threshold and the within-pair difference in threshold. This is consistent with figure 4b and suggests that a positive social drift rate is beneficial to members with lower thresholds. Finally, for pair members with higher thresholds (right column), the optimal social drift rates decrease as a function of the within-pair threshold in a pair approaching zero (i.e. no reliance on social information) for large within-pair differences in thresholds. If the higher-threshold member is much more accurate, they should stop relying on their partner’s choice, especially because they will also be exposed to their partner’s inferior information for a relatively long period owing to the large differences in response time. This pattern was independent of the threshold of the lower-threshold member. These results match the empirical results shown in figure 4a (right panel of scatter plots).

Although the mean accuracy of a pair and the accuracy of the pair’s leader depended only on the lower-threshold member (figure 3e,g), accuracy in the social condition was not worse than in the personal condition (figure 3a). The simulation results suggest two mechanisms that prevented pairs from becoming less accurate: lower-threshold members increased their thresholds in the social condition and became more accurate (electronic supplementary material, Supplementary Results and figure S4) and higher-threshold members incorporated less social information.

## 3. Discussion

Although speed–accuracy trade-offs are fundamental to almost all decision-making contexts [1], previous research on human group decision-making has largely ignored the role of individual differences in speed–accuracy trade-off preferences. We therefore combined a real-time interactive study with modelling and simulating individual differences in decision conservativeness, perceptual sensitivity and social information use. Our study produced two major findings.

First, pair members with lower thresholds in the personal condition made decisions earlier (i.e. became the leader) in the social condition (figure 3c), thereby limiting the pair’s mean accuracy (figure 3g). This result is consistent with previous studies showing that earlier decisions affect later ones in sequential group decision-making in humans [15–19] and other animal groups [31,32]. Pair members’ perceptual sensitivity, as indexed by personal drift rate, did not affect the pair’s mean accuracy (figure 3h). Individual differences in speed–accuracy trade-off preference can therefore be detrimental to sequential decision-making, since less accurate members may trigger chains of less accurate information that may be difficult for more accurate members to correct. Future work could test whether similar mechanisms operate in real-world contexts, such as collective attention [33], consumer choices in online markets [34] and risk communication in medical treatments [35]. Future work could also investigate whether the effects of initial biases can be amplified in sequential decision-making, because individuals with more extreme initial biases have been predicted to decide earlier [21]. Our results contrast with earlier work showing that more confident or knowledgeable members decide first in sequential group decisions, leading to better social decisions [15,17]. These differences may be the result of different group sizes—these previous studies used substantially larger groups—or experimental stimuli. In our task, decision speed was mostly governed by threshold (and not by personal drift rate); social interactions may be more beneficial in tasks in which decision speed is also a function of personal drift rate.

Although the parameter recovery criteria were met reasonably well (electronic supplementary material, figure S1; [36]), the recovery performance was not particularly high for the social drift rate for lower-threshold members (electronic supplementary material, figure S7) owing to the small number of trials where they saw social information (especially when paired with a member with a much higher threshold). In such situations with few data points, individual estimates converge to the population mean (known as ‘shrinkage’ of parameters), since we assumed a hierarchical structure of the parameter. However, this shrinkage cannot explain the effect that lower-threshold members have higher social drift rates (left panel of figure 4a), as its effect goes in the opposite direction; that is, it will lead to an underestimation of the social drift rate of the lower-threshold members. Future studies with more trials are needed to overcome this limitation.

Second, pair members adjusted their social information use depending on whether they had the higher or lower threshold in their pair (figure 4a). Although accuracy did not improve in the social condition (figure 3b), simulation analyses suggest that pairs used social information adaptively (figure 4b). This finding is consistent with previous studies showing that individuals can adjust their weighting of social information depending on its accuracy [25–27]. Future research could directly investigate the processes of learning the quality of social information and adaptive weight adjustments, for example, by using a reinforcement learning framework. We also tested for a change in social drift rate over trials but found no evidence for this (electronic supplementary material, figure S8). However, in many contexts, individuals may change their weights on social information, depending on their evolving assessment of the quality of their partner’s responses compared with their own. Here the learning environment is likely a key moderator. In our design, participants received feedback after each trial, making it relatively easy for them to learn their accuracy as well as that of their partners. In situations without such feedback (e.g. [37]), adaptive social weighting may be more challenging and negative effects of individual differences in speed–accuracy trade-off preference may be even more pronounced. Furthermore, earlier social information could also be seen as more reliable and impactful in some cases [38,39]. Note, however, that in our modeling approach with a constant social drift rate, social information that arrives earlier will also have more influence because the other member is exposed to it for longer.

By using a constant social drift rate, we assumed that social information accumulates gradually over time (figure 1c). This approach has been successfully used to model social dynamics; for example, previous studies were able to recover the empirical social dynamics in groups of up to 17 individuals [15] and in groups faced with a situation where one option is chosen more quickly than the other [40]. However, it is also possible that social information impacts an individual’s accumulation process only once by an instantaneous (and not gradual) increase in evidence [21,38,39,41]. Modelling both gradual and instantaneous processes is promising (e.g. [16]), but this was not feasible in the current setting, where the number of trials is not large enough to estimate the parameters. Future studies are needed to understand whether one or the other of these processes is more important for collective behaviour and under which conditions, or whether they are equally significant.

The DDM has been instrumental in revealing human cognitive processes in a wide variety of real-world contexts, including perception [42], purchasing [43], altruism [44] and police shootings [45]. Almost all work in real-world contexts has focused on individual decision-making—yet humans rarely make decisions alone. Instead, humans often make decisions under social influence [18], which can amplify individual biases and result in group polarization (e.g. information cascades [20], economic bubbles [46], superstar phenomena in product popularity [34] and collective rule-breaking [47]). There is ample opportunity to investigate the dynamics in real-world contexts through the lens of the DDM by, for instance, testing how individual differences in speed–accuracy trade-off preferences affect who decides first, how this information is transmitted through groups and how individuals in turn adjust their cognitive architecture. To better understand and tackle collective human behaviour, future studies would benefit from focusing on interactive situations, combined with cognitive modeling [48,49].

## 4. Methods

### (a) Participants

We recruited 114 participants without colour vision deficiency online using Prolific (60 females, 50 males, and four non-binary; mean age ± s.d. = 32.3 ± 7.3 years; age range = 20−45 years). Only individuals from the United Kingdom who used a desktop or laptop and had a Prolific approval rating greater than 90% were allowed to participate.

The number of participants was determined based on previous studies on dyadic decision-making (e.g. [22,50]). The duration of a session was set at around 30 min; the number of trials—76 per condition—was determined accordingly. See ‘Data exclusion’ in electronic supplementary material for data exclusion criteria.

### (b) Experimental procedure

#### (i) Overview

Participants started with 76 trials in a personal condition, followed by another 76 in a social condition. In the personal condition, participants completed the task alone. In the social condition, they completed the task with another participant while simultaneously seeing each other’s answers in real time. Participants then completed a post-session questionnaire (electronic supplementary material, Supplementary Methods) and received a £4 base fee plus a bonus (range: £1.20–£2.80), resulting in an hourly wage of approximately £12.

#### (ii) Task

We used a modified version of the task and stimuli in [51]. In the personal condition, participants observed a sequence of dot stimuli for up to 10 s (figure 1a). Each stimulus lasted for 0.33 s and contained a mix of 40 red and blue dots. While the stimuli were presented (i.e. within the 10 s), participants had to identify the dominant colour. If the participant did not answer within 10 s, the stimuli disappeared and an extra 3 s countdown timer appeared to prompt a response in order to minimize the number of missed trials. Participants chose their colour by pressing the left- or right-arrow key. The key–colour combination was counterbalanced across participants. At the end of each trial, participants received feedback on their answer. They earned 100 points (= £0.02) for a correct response, minus 2 points per second they took to answer; they earned nothing for a wrong response. The small time cost was introduced to prevent participants from waiting until the last second for their partner’s response in the social condition [15]. Before the personal condition, all participants passed comprehension quizzes and completed eight practice trials, which were excluded from the analyses.

In the social condition, participants were anonymously paired with another participant who had also finished the personal condition. In pairs, they performed the same task as in the personal condition and observed the same sequence of stimuli in a given trial. They also received social information—namely, their partner’s choice—in real time (‘Social information’ in figure 1a). Participants could not alter their decision once made. Both participants were shown their partner’s choice in real time. Participants’ rewards were solely based on their individual performance and participants were told that they were not in competition with each other.

#### (iii) Stimuli

The 40 red and blue dots (radius: 6 pixels) appeared randomly in the centre of a white circle (radius: 200 pixels) such that they did not overlap (figure 1a). In half of the trials in each condition, the correct option was red (*M* = +4); in the other half it was blue (*M* = −4). The number of red minus blue dots followed a truncated normal distribution with *M* = ± 4 and s.d. = 16 lying within ±40, and was rounded to the nearest even number. These settings were chosen to elicit an individual accuracy of approximately 75%. The trial order was pseudorandomized such that there were no more than five consecutive trials of the same correct answer. In the social condition, the correct answer was identical for both pair members, since they observed the same stimuli.

Red and blue circles were presented on the left and right sides of the screen, respectively, to remind participants which keys (left or right arrow) corresponded to the options. A participant’s choice was indicated with a white square around their selected colour; their partner’s choice was indicated with a filled circle (‘Social information’ in figure 1a). Whenever a participant made a decision, the stimuli of coloured dots disappeared for them (but not for their partner). The experiment was programmed using oTree [52].

### (c) Analyses

#### (i) Regression analyses

See electronic supplementary material, Supplementary Methods and table S2 for the details of the regression models (probability distributions, variables, model formulas, random-effect structures).

#### (ii) The DDM and SDDM

The DDM for the personal condition (figure 1b) had four parameters: decision threshold (*θ*), non-decision time (*τ*, in seconds), initial bias (*β*) and personal drift rates toward correct options: *δ*_red_ or *δ*_blue_ if red or blue was the correct option, respectively. The decision threshold represents the distance between the two decision boundaries, with one boundary at zero (choosing red) and the other at *θ* (choosing blue). The non-decision time reflects the initial duration that passed without any evidence accumulation (e.g. motor response). To obtain reasonable estimates, the non-decision time was constrained between 0 and 1 s, following previous research (e.g. [53,54]). The initial bias represents where evidence accumulation started between the thresholds (e.g. prior expectation or preference for one option over the other). The initial bias was fixed at 0.5, exactly in the middle of the boundaries, because participants had no information about the correct answer before seeing the stimuli. Personal drift rates capture the absolute trend of the accumulation toward the correct options. Given these parameters, the evidence accumulation follows the stochastic process $\frac{d}{dt}{Χ}_{t}∼Normal(\delta ,\sigma^{2})$ with the scaling parameter σ fixed to 1. The decision time is modelled as the first time at which evidence hits either boundary.

The SDDM for the social condition (figure 1c) had an additional parameter: social drift rate (*δ*_*s*ocial_). The social drift rate reflects an additional drift toward a partner’s chosen option. Thus, the drift rate (*δ*) after the presentation of social information at a trial is:


(4.1) $$\delta =\left\{ \begin{array}{l} \begin{array}{rlrl} \delta_{\text{blue}} & +\delta_{\text{social}}, & & \text{if }a=+1,s=+1,\\ \delta_{\text{blue}} & -\delta_{\text{social}}, & & \text{if }a=+1,s=-1,\\ -(\delta_{\text{red}} & +\delta_{\text{social}}), & & \text{if }a=-1,s=-1,and,\\ -(\delta_{\text{red}} & -\delta_{\text{social}}), & & \text{if }a=-1,s=+1, \end{array} \end{array}\right.$$

where *a* is the correct answer (blue = +1, red = −1) and *s* is the social information in the trial (blue = +1, red = −1). Note that *δ*_red_ is an absolute personal drift rate toward the lower boundary. Therefore, *δ*_red_ is multiplied by a minus sign in equation (4.1). The average of *δ*_blue_ and *δ*_*r*ed_ was used as an index of the participant’s personal drift rate.

The DDM was fitted to the response time distributions of red or blue choices in the personal condition. The SDDM was fitted to the response time distributions in the social condition. We used data from all trials, regardless of whether the participant responded first or second in the trial.

## Data Availability

The experimental materials, data and analysis codes that support the findings of this study are available on the Open Science Framework [55]. Supplementary material is available online [56].
